# The Association Between Pulmonary Hypertension, End-Stage Kidney Disease, and Death: An Analysis of the Chronic Renal Insufficiency Cohort Study (CRIC)

**DOI:** 10.1016/j.xkme.2026.101399

**Published:** 2026-05-12

**Authors:** Marcelle Tuttle, Hocine Tighiouart, Tatsufumi Oka, Wendy McCallum, Sankar D. Navaneethan, Steven M. Kawut, Mark J. Sarnak

**Affiliations:** 1 Tufts Medical Center, Boston, MA; 2 Tufts Clinical and Translational Science Institute, Tufts University, Boston, MA; 3 Institute for Clinical Research and Health Policy Studies, Tufts Medical Center, Boston, MA; 4 Department of Nephrology, Osaka University Graduate School of Medicine, Suita, Osaka, Japan; 5 Baylor College of Medicine and Michael E. DeBakey VA Medical Center, Houston, TX; 6 Perelman School of Medicine, University of Pennsylvania, Philadelphia, PA

**Keywords:** Chronic kidney disease, Chronic Renal Insufficiency Cohort Study (CRIC), end-stage kidney disease, kidney failure with replacement therapy, pulmonary hypertension

## Abstract

**Rationale & Objective:**

Pulmonary hypertension (PH) is highly prevalent in patients with chronic kidney disease and has been shown to be associated with an increased risk of death and cardiovascular disease in prior studies; however, its association with kidney outcomes is less clear. We evaluated the association between PH and tricuspid regurgitation velocity (TRV) with incident kidney failure with replacement therapy (KFRT) and secondarily death.

**Study Design:**

Longitudinal prospective cohort study.

**Setting & Participants:**

2,419 participants from the Chronic Renal Insufficiency Cohort Study (CRIC).

**Exposures:**

PH, defined as a TRV >2.8 m/s on echocardiograms, and also using TRV as a continuous variable.

**Outcomes:**

KFRT, death before and after KFRT.

**Analytical Approach:**

Time-updated Cox proportional hazards model using multiple TRV measurements per participant adjusted for demographics, comorbid conditions, medications, laboratory values, and echocardiographic features.

**Results:**

At baseline, 343 participants (14.2%) had PH and 134 (5.5%) developed PH after 7 years of serial echocardiograms. Over a median follow-up duration of 12.1 years, 751 (31.0%) reached KFRT and over 13.1 years 978 (40.4%) died. PH was associated with a 60% higher risk of KFRT when compared with participants who did not have PH (HR, 1.60; 95% CI, 1.33-1.92) and a 68% higher risk of death (HR, 1.68; 95% CI, 1.45-1.95). Each 1 SD higher TRV was associated with 20% higher risk of KFRT (HR, 1.20; 95% CI, 1.12-1.28) and a 23% higher risk of death (HR, 1.23; 95% CI, 1.16-1.30).

**Limitations:**

Use of TRV as a proxy to define PH rather than right heart catheterization.

**Conclusions:**

PH is associated with an increased risk of KFRT and death in participants with chronic kidney disease.

## Plain-Language Summary

High pressure in the pulmonary arteries, which supply blood to the lungs, also known as pulmonary hypertension, is a rare disease in the general population, but it is common among patients with chronic kidney disease. When it is found in chronic kidney disease patients, it has been shown to be associated with higher rates of death. Prior reports have differed as to whether pulmonary hypertension is also associated with kidney failure. To answer this question, we used the Chronic Renal Insufficiency Cohort Study (CRIC), the longest running cohort study of participants with chronic kidney disease. In this study, we show that pulmonary hypertension is associated with higher risk of kidney failure, death, and a composite of kidney failure or death.

Pulmonary hypertension (PH) is defined as mean pressure in the pulmonary arteries >20 mm Hg.^1^ It is commonly found in patients with chronic kidney disease (CKD) with increasing prevalence as kidney function declines, ranging from ∼6% of people with CKD stage 1 up to ∼33% in people with CKD stage 5.^1^^,^^2^ Patients with CKD and prevalent PH are at increased risk of death and cardiovascular disease when compared with those without PH.^2^, ^3^, ^4^, ^5^, ^6^

However, it is less clear whether PH is associated with kidney outcomes. Increased right-sided cardiac pressures are associated with declines in kidney function in some heart failure studies,^7^, ^8^, ^9^, ^10^, ^11^ potentially through a mechanism of increased renal vein pressures. It is reasonable to hypothesize that PH would be associated with faster kidney disease progression through the same mechanism, but the data have not been consistent. In a longitudinal cohort study of 2,959 participants with CKD in the Chronic Renal Insufficiency Cohort Study (CRIC), prevalent PH (defined as a pulmonary artery systolic pressure [PASP] >35 mm Hg or tricuspid regurgitation velocity [TRV] >2.5 m/s) was not significantly associated with kidney outcomes (defined as a decline in estimated glomerular filtration rate [eGFR] of 50% or kidney failure with replacement therapy [KFRT]) in fully adjusted models.^2^ Similarly, a case-control study consisting of Medicare claims data demonstrated no association between PH on claims data and KFRT for those with longer durations of follow-up.^3^ Conversely, a large retrospective cohort study of patients who underwent clinical echocardiograms demonstrated that higher estimated pulmonary artery pressures were associated with a composite kidney outcome of 50% eGFR decline and KFRT.^12^

Given the inconsistencies in the literature, the fact that additional echocardiograms were performed in CRIC participants that were not incorporated into prior analyses, the threshold for TRV to be indicative of likely PH has risen with new definitions of PH, as well as longer follow-up with more KFRT events in CRIC participants since the initial analyses, we evaluated whether participants with prevalent PH or who develop PH are at higher risk for KFRT than those without PH using time-updated analyses.^1^ Secondarily, we examined the relation of PH with death and a composite of death and KFRT.

## Methods

### Study Population

The study population was derived from CRIC, an ongoing longitudinal study of 3,939 participants with CKD. Starting in 2003, adult participants with eGFRs of 20-70 mL/min/1.73 m^2^ using the Modification of Diet in Renal Disease equation were enrolled and have been continuously followed. Procedures of the study were previously described.^13^^,^^14^

Participants were included in the present study if they had ≥1 measurement of TRV before reaching KFRT. One participant was missing information regarding the timing of their echocardiograms and was excluded from the study sample. Echocardiograms were scheduled as a routine part of CRIC at the year 1, 4, and 7 visits. Echocardiographic data were excluded if they were obtained as part of CRIC-Plus, an ancillary study of echocardiograms in CRIC participants with eGFR <20 mL/min/1.73 m^2^. We excluded these echocardiograms to avoid bias in that these participants had additional TRV measurements and thereby more opportunity to meet criteria for our main exposure of interest. Echocardiograms performed after the participants had initiated dialysis were also excluded as it was anticipated that the volume shifts that occur during dialysis may change TRV measurements and decrease their accuracy.

All study participants of CRIC provided written informed consent. Ethics approval for this specific analysis was obtained from the Tufts Medical Center Institutional Review Board. The primary source of data for the study, apart from the TRV variable, was from the National Institute of Diabetes and Digestive and Kidney Diseases biorepository under CRIC release version 12. The TRV variable was obtained directly from the University of Pennsylvania CRIC data coordinating center and was measured for a prior study.^2^

### Echocardiogram Procedures

All echocardiograms were obtained at each individual study center and read at the University of Pennsylvania core laboratory. TRV was obtained via continuous wave doppler of the right ventricular inflow tract using the parasternal, apical, and subcostal imaging windows, and left ventricular ejection fraction was calculated via digitization of the left ventricular endocardial borders of apical 4 chamber images at end-diastole and end-systole. Right atrial pressure was estimated by visual inspection of the inferior vena caval diameter through several respiratory cycles and added to the right ventricular pressure gradient obtained from the TRV to ascertain the PASP. This was then calculated as the stroke volume, divided by the end-diastolic volume multiplied by 100%. Left ventricular diastolic dysfunction was graded as normal, mildly abnormal, moderately abnormal, and severely abnormal as previously described using the ratio of E:A wave velocities, the mitral valve deceleration time, and the ratio of systolic:diastolic pulmonary vein flow.^15^ To generate intraclass correlation statistics, a 2% random sample of the echocardiograms were read each year. These were 0.97 for TRV and 0.854 for left ventricular ejection fraction.^2^

### Exposures

The primary exposure of interest was PH, defined as a TRV >2.8 m/s, a commonly used threshold for echocardiographic screening for the diagnosis of PH (PH definition 1).^16^, ^17^, ^18^ As TRV alone may underestimate pulmonary artery pressures, we performed a secondary analysis using a definition of PH as TRV >2.8 m/s or PASP >35 mm Hg in those CRIC participants who had PASP available (PH definition 2).^18^ As a third exposure, TRV was examined as a continuous variable and using quartiles. For the primary analysis, PH status and TRV were examined as time-updated exposures such that for each participant, every reading of PH status and TRV were incorporated into the models.

### Outcomes

The main outcome of interest was KFRT, defined as start of dialysis or receipt of a kidney transplant. KFRT was adjudicated as previously described through biannual interviews of participants, review of medical records of all hospitalizations, and linkage with the US Renal Data System.^19^ As a secondary outcome, death uncensored for KFRT status was examined, which was determined via interviews of next of kin, review of hospitalization records, and social security death vital status records. As there is a high competing rate of death in patients with CKD, a composite outcome of KFRT or death before KFRT was also examined.

### Covariates

All covariates were derived at the time of or before the first echocardiogram for each subject. Laboratory data and comorbid conditions were obtained at prespecified study visits. Models were adjusted serially for (1) age, sex, diabetes, smoking, systolic blood pressure, history of cardiovascular disease, hypertension, chronic obstructive pulmonary disease, cancer, use of angiotensin-converting enzyme inhibitors and/or angiotensin receptor blockers, aldosterone antagonists, diuretics, and statins, and left ventricular ejection fraction and (2) the same covariates as model 1 with the addition of eGFR and 24-hour urinary albumin excretion. As left ventricular diastolic dysfunction is a key risk factor for the development of PH in patients with CKD, we performed a sensitivity analysis with additional adjustment for left ventricular diastolic dysfunction (model 3) and also evaluated an interaction term between left ventricular diastolic dysfunction and the exposure of interest with KFRT, death, and a composite (model 4).

### Missing Data

In the study sample, 41 participants (1.7%) were missing baseline ejection fraction, 266 (11.0%) were missing a baseline assessment of left ventricular diastolic dysfunction, 95 (3.9%) were missing baseline 24-hour urinary albumin, 2 (0.08%) had missing information regarding whether they had chronic obstructive pulmonary disease, and 1 (0.04%) was missing baseline medication data. For the primary analyses, these missing values were imputed using multiple imputation and predictive mean matching by including all the variables of interest and adjustment covariates in the model over 20 imputations. As a sensitivity analysis, the complete case was also examined.

Additionally, PASP was missing in 472 of the 3,686 echocardiograms included in the study sample. To avoid imputing the exposure of interest, for the analyses of PH definition 2, the patient population was restricted to those with PASP values.

### Statistical Analyses

To include each measurement of TRV for each participant to enable assessment of PH status over time, time-updated Cox proportional hazards models were used to examine the relation between PH and the outcomes of interest (Fig S1). PH status was assessed at the time of each echocardiogram, and time at risk was specified to start at the time each TRV measurement available for each participant and to end at the next available TRV measurement (if available), the outcome of interest, or censoring. To account for the competing risk of death to the development of KFRT, Fine-Gray subdistribution hazard models were also performed.

To assess for nonlinear relationships, adjusted models using restricted cubic splines with 4 knots were used to model the relation between TRV and each outcome of interest in a continuous fashion, and plots were generated with each covariate fixed at the mean. Each TRV value was assigned to a quartile based on the limits of the quartiles for TRV values at baseline.

We also examined whether rates of change in TRV and incident PH were associated with KFRT and death in individuals with ≥2 TRV measurements. Rate of change of TRV was defined as (last TRV measurement – first TRV measurement) / years between echocardiograms. Incident PH was first as a TRV ≤2.8 m/s on the first echocardiogram and a TRV >2.8 m/s on the last echocardiogram. The second definition defined incident PH as a TRV ≤2.8 m/s and PASP ≤35 mm Hg on the first echocardiogram and a TRV >2.8 m/s or PASP >35 mm Hg on the last echocardiogram. Participants with incident PH were compared with those participants who never developed PH by these definitions. Analyses were performed using multiple imputation and via the complete case. Models were serially adjusted for the same characteristics as the primary analyses with covariates obtained at or before the time of the second echocardiogram (the start of time at risk for this model) with the addition of an adjustment for baseline TRV.

## Results

### Baseline Characteristics of the Study Sample

**Figure 1:**
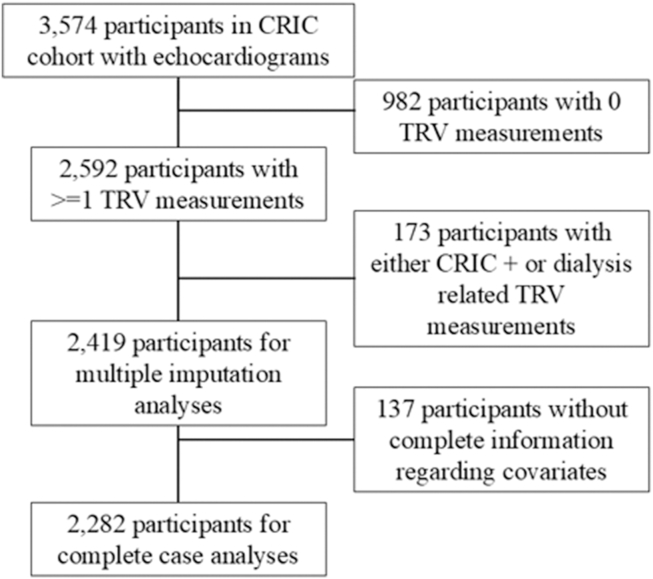
Study flow diagram. Abbreviations: CRIC, Chronic Renal Insufficiency Cohort Study; TRV, tricuspid regurgitation velocity.

Of the 3,574 participants in CRIC with echocardiograms, 2,592 participants had one or more measurements of TRV. When compared with those without measured TRV signals, those with one or more TRV measurements were older, more likely to be female, and had lower rates of cardiovascular disease and smoking (Table S1). Of the participants with one or more TRV measurements, 173 had only CRIC-Plus or TRV measurements that occurred after KFRT and were excluded, leaving 2,419 for analysis (Fig 1). At baseline, 343 (14.2%) had PH and 134 (5.5%) developed PH on subsequent echocardiograms. Of those who had PH at baseline, 83 (3.4%) had ‘regression’ of their PH at any time point afterward, meaning that they no longer met the criteria for PH on any subsequent echocardiogram. Baseline characteristics by PH status are displayed in Table 1. Overall, those with PH at baseline had higher systolic blood pressures, higher prevalence of diabetes, hypertension, chronic obstructive pulmonary disease, and cardiovascular disease, higher use of diuretics and statins, and higher levels of albuminuria but lower eGFRs. When examined per quartile of TRV, similar trends were observed (Table S2).

**Table 1:** Baseline Characteristics by Baseline PH Status Using a Definition of TRV >2.8 m/s

Patient Characteristic	Total	No PH	PH	*P* value
n	2,419	2,076	343	
**Demographics**				
Age, y	60.5 (10.6)	60.1 (10.8)	63.4 (9.0)	<0.001
Women	1,133 (46.8%)	976 (47.0%)	157 (45.8%)	0.71
Race				<0.001
Non-Hispanic White	1,005 (41.5%)	902 (43.4%)	103 (30.0%)	
Non-Hispanic African American	989 (40.9%)	825 (39.7%)	164 (47.8%)	
Hispanic	332 (13.7%)	267 (12.9%)	65 (19.0%)	
Other	93 (3.8%)	82 (3.9%)	11 (3.2%)	
**Vitals**				
Systolic blood pressure, mm Hg	127.3 (21.7)	125.9 (20.7)	135.8 (25.1)	<0.001
**Comorbid conditions**				
Chronic obstructive pulmonary disease	140 (5.8%)	112 (5.4%)	28 (8.2%)	0.06
Diabetes	1145 (47.3%)	929 (44.7%)	216 (63.0%)	<0.001
Hypertension	2164 (89.5%)	1837 (88.5%)	327 (95.3%)	<0.001
Cardiovascular disease^a^	881 (36.4%)	704 (33.9%)	177 (51.6%)	<0.001
Active smoking	272 (11.2%)	241 (11.6%)	31 (9.0%)	0.19
Cancer	245 (10.1%)	212 (10.2%)	33 (9.6%)	0.81
**Laboratory values**				
eGFR, mL/min/1.73 m^2^	42.0 (16.1)	42.9 (16.3)	36.3 (13.7)	<0.001
Urinary albumin excretion, g/24 h	0.04 [0.01-0.39]	0.04 [0.01-0.34]	0.13 [0.02-1.19]	<0.001
**Medications**				
ACE inhibitors/ARBs	1,639 (67.8%)	1,404 (67.6%)	235 (68.7%)	0.74
Aldosterone antagonist	95 (3.9%)	76 (3.7%)	19 (5.6%)	0.13
Diuretics	1,409 (58.3%)	1162 (56.0%)	247 (72.2%)	<0.001
Statins	1,467 (60.7%)	1219 (58.7%)	248 (72.5%)	<0.001
**Echocardiogram variables**				
Left ventricular ejection fraction, %	53.2 (9.2)	53.7 (8.5)	50.4 (12.2)	<0.001
Baseline TRV, cm/s	246.0 (35.1)	235.5 (22.6)	309.9 (29.0)	<0.001

*Note:* Data shown as n (%), mean (SD), or median [25th, 75th percentiles].

Abbreviations: ACE, angiotensin-converting enzyme; ARB, angiotensin receptor blocker; eGFR, estimated glomerular filtration rate; PH, pulmonary hypertension; TRV, tricuspid regurgitation velocity.

a Cardiovascular disease is defined as any of myocardial infarction/revascularization, congestive heart failure, stroke, or peripheral vascular disease.

### Risk of KFRT

**Figure 2:**
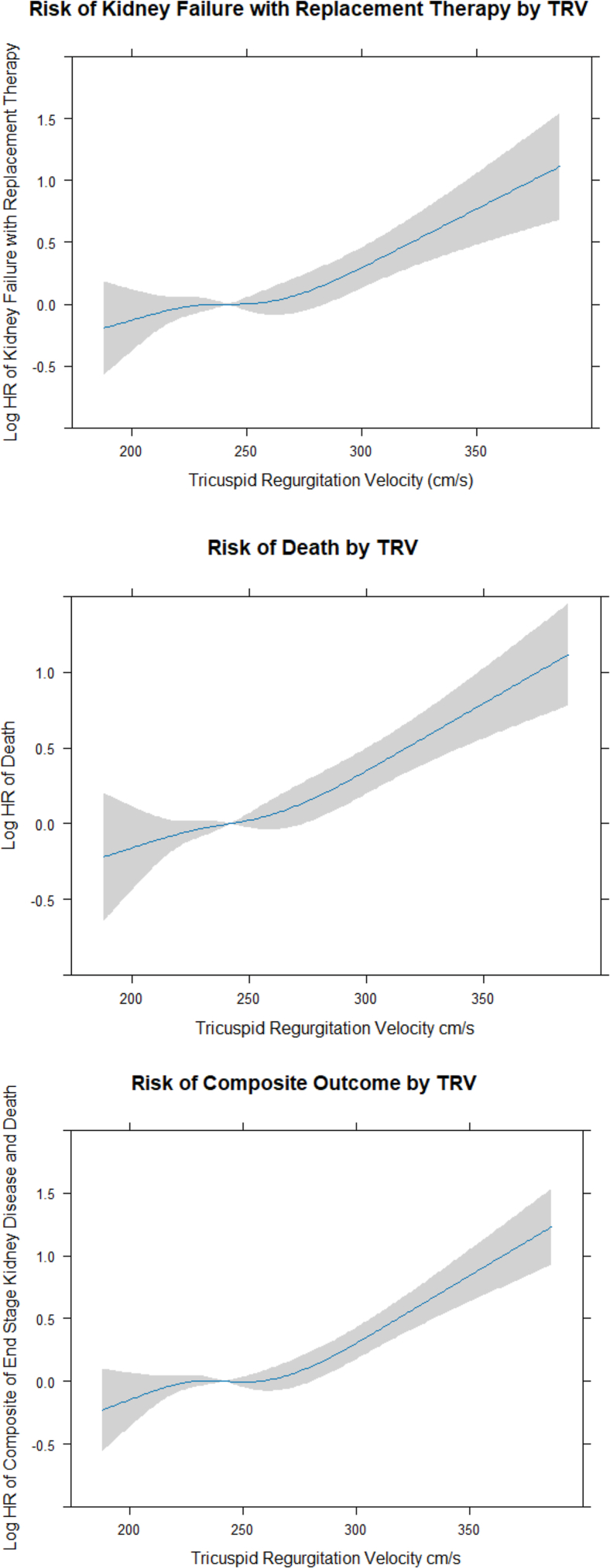
Continuous association of time-updated TRV and kidney failure with replacement therapy, death, and a composite. Graphs demonstrate the association between TRV and outcomes using restricted cubic splines with 4 knots to investigate for nonlinearity with adjustments for age, sex, diabetes, hypertension, chronic obstructive pulmonary disease, cancer, history of cardiovascular disease, smoking, systolic blood pressure, use of angiotensin-converting enzyme inhibitors/angiotensin receptor blockers, aldosterone antagonists, statins, and diuretics, ejection fraction, estimated glomerular filtration rate, and 24-hour urinary albumin excretion. Average HR is displayed via the continuous line with the 95% confidence interval represented by the shading. Kidney failure with replacement therapy *P* value for nonlinearity = 0.09. Death *P* value for nonlinearity = 0.17. Composite *P* value for nonlinearity = 0.01. Abbreviations: HR, hazard ratio; TRV, tricuspid regurgitation velocity.

Over a median follow-up duration of 12.1 (interquartile range 8.7, 13.7) years, 751 (31.0%) reached KFRT. PH was associated with a 60% (hazard ratio [HR], 1.60; 95% confidence interval (CI), 1.33-1.92) increased risk of KFRT in adjusted analyses (Table 2), with similar results observed when the second definition of PH was used. Higher TRV was also associated with increased risk of KFRT (HR, 1.20; 95% CI, 1.12-1.28 for each standard deviation [SD] higher TRV) with highest risk in the fourth quartile, although without any evidence of nonlinearity (Fig 2). Similar relationships were observed when the complete case was used rather than multiple imputation (Table S3).

**Table 2:** Association of Time-Updated PH Status and TRV With Kidney Failure With Replacement Therapy, Death, and a Composite of Kidney Failure With Replacement Therapy and Death

Kidney Failure With Replacement Therapy							
	N	N Events	Follow-up (y)	Event Rate per 100-py	Unadjusted HR (95% CI)	Model 1 HR (95% CI)	Model 2 HR (95% CI)
**PH**							
PH definition 1	3,686	751	20,503	3.66	2.20 (1.85-2.62)	1.74 (1.45-2.09)	1.60 (1.33-1.92)
PH definition 2	3,214	680	18,439	3.69	2.16 (1.81-2.57)	1.69 (1.41-2.03)	1.62 (1.34-1.95)
**TRV**							
Continuous per SD of TRV	3,686	751	20,503	3.66	1.35 (1.26-1.44)	1.24 (1.15-1.32)	1.20 (1.12-1.28)
Quartile 1	919	175	5,803	3.02	Ref	Ref	Ref
Quartile 2	848	151	4,869	3.10	1.01 (0.82-1.26)	0.97 (0.78-1.20)	0.95 (0.76-1.19)
Quartile 3	933	166	5,338	3.11	1.02 (0.83-1.27)	0.93 (0.75-1.16)	0.97 (0.78-1.21)
Quartile 4	986	259	4,493	5.77	1.86 (1.54-2.26)	1.48 (1.21-1.82)	1.43 (1.16-1.75)
**Death**							
**PH**							
PH definition 1	3,686	978	24,486	3.99	2.70 (2.34-3.11)	1.82 (1.57-2.11)	1.68 (1.45-1.95)
PH definition 2	3,214	844	22,110	3.82	2.56 (2.21-2.98)	1.77 (1.52-2.07)	1.63 (1.39-1.91)
**TRV**							
Continuous per SD of TRV	3,686	978	24,486	3.99	1.48 (1.41-1.56)	1.27 (1.20-1.34)	1.23 (1.16-1.30)
Quartile 1	919	175	6,759	2.59	Ref	Ref	Ref
Quartile 2	848	198	5,681	3.49	1.37 (1.12-1.68)	1.18 (0.96-1.45)	1.19 (0.97-1.45)
Quartile 3	933	210	6,243	3.36	1.31 (1.07-1.60)	1.03 (0.84-1.26)	1.04 (0.85-1.27)
Quartile 4	986	395	5,803	6.81	2.68 (2.25-3.21)	1.73 (1.44-2.08)	1.64 (1.36-1.97)
**Composite of Kidney Failure With Replacement Therapy or Death**							
**PH**							
PH definition 1	3,686	1,352	20,503	6.59	2.44 (2.15-2.78)	1.79 (1.57-2.04)	1.60 (1.40-1.84)
PH definition 2	3,214	1,186	18,439	6.43	2.31 (2.03-2.64)	1.74 (1.52-1.99)	1.57 (1.37-1.81)
**TRV**							
Continuous per SD of TRV	3,686	1,352	20,503	6.59	1.43 (1.37-1.50)	1.27 (1.20-1.33)	1.21 (1.15-1.27)
Quartile 1	919	278	5,803	4.79	Ref	Ref	Ref
Quartile 2	848	286	4,869	5.87	1.23 (1.04-1.45)	1.12 (0.95-1.32)	1.10 (0.93-1.30)
Quartile 3	933	298	5,338	5.58	1.17 (0.99-1.37)	1.00 (0.85-1.18)	0.99 (0.84-1.17)
Quartile 4	986	490	4,493	10.91	2.28 (1.97-2.64)	1.65 (1.41-1.92)	1.49 (1.28-1.74)

*Note:* Model 1 is adjusted for age, sex, diabetes, hypertension, chronic obstructive pulmonary disease, cancer, history of cardiovascular disease, smoking, systolic blood pressure, use of angiotensin-converting enzyme inhibitors/angiotensin receptor blockers, aldosterone antagonists, statins, and diuretics, and ejection fraction. Model 2 is adjusted for the same factors as model 1 and estimated glomerular filtration rate and 24-hour urinary albumin excretion.

Abbreviations: CI, confidence interval; HR, hazard ratio; PH, pulmonary hypertension; py, person-years; Ref, reference; SD, standard deviation; TRV, tricuspid regurgitation velocity.

### Death

Over a median follow-up duration of 13.1 (interquartile range 11.9, 14.3) years, 978 participants (40.4%) died. PH was associated with a 68% (HR, 1.68; 95% CI, 1.45-1.95) increased risk of death (Table 2). TRV was also associated with increased risk of death (HR, 1.23; 95% CI, 1.16-1.30 for each SD higher TRV) with the highest risk in the fourth quartile. No significant nonlinearity was observed (Fig 2). Results from the complete case analysis were similar to those used in the multiple imputation (Table S3).

### Composite Outcome

1,352 (55.9%) progressed to KFRT or death over a median follow-up duration of 13.1 (interquartile range 8.7, 13.7) years. A total of 601 deaths occurred before participants reached KFRT. Both PH and higher TRV were associated with an increased risk of the composite outcome (Table 2). This relation was found to be nonlinear with much of the association driven by the highest quartile of TRV (Fig 2).

### Left Ventricular Diastolic Dysfunction

We found neither attenuation of our results nor significant interaction between any of our exposures of interest and diastolic dysfunction (Table S4).

### Fine-Gray Subdistribution Hazard Models

When the competing risk of death was taken into account, PH and TRV remained significantly associated with an increased risk of KFRT (subdistribution HR, 1.38; 95% CI, 1.16-1.63 and subdistribution HR, 1.08; 95% CI. 1.02-1.15, respectively) (Table S5).

### Incident PH analysis

Of the participants, 1,076 had ≥2 TRV measurements before reaching dialysis (Fig S2). Over a median period of 3.0 (interquartile range 2.9, 3.7) years between echocardiograms, 119 participants developed incident PH and 819 never developed PH. Baseline characteristics of the subcohort by PH status and quartile of rate of TRV change are displayed in Tables S6 and S7.

Incident PH was associated with a 69% (HR, 1.69; 95% CI, 1.13-2.53) increased risk of KFRT (Table 3). For every SD higher rate of TRV change, there was a 33% increase in the hazard of KFRT (HR, 1.33; 95% CI, 1.17-1.52). Similar results were obtained when the complete case was examined (Table S8).

**Table 3:** Association of Incident Pulmonary Hypertension/Rate of TRV Change With Kidney Failure With Replacement Therapy, Death, and a Composite

Exposure	N	N Events	Follow-up (y)	Event Rate per 100-py	Unadjusted HR (95%CI)	Model 1 HR (95%CI)	Model 2 HR (95%CI)	Model 3 HR (95%CI)
**Kidney Failure With Replacement Therapy**								
PH definition 1	938	210	6,814	3.08	2.21 (1.56-3.14)	2.10 (1.47-3.01)	2.03 (1.39-2.97)	1.69 (1.13-2.53)
PH definition 2	733	176	5,328	3.30	2.31 (1.59-3.35)	2.23 (1.53-3.24)	2.09 (1.41-3.09)	1.96 (1.27-3.01)
Continuous per SD increase in rate of TRV	1,076	259	7,559	3.43	1.14 (1.00-1.31)	1.38 (1.21-1.58)	1.33 (1.15-1.55)	1.33 (1.17-1.52)
Quartile 1	269	75	1,826	4.11	Ref	Ref	Ref	Ref
Quartile 2	269	53	2,096	2.53	0.63 (0.44-0.90)	0.82 (0.56-1.20)	0.75 (0.52-1.10)	0.75 (0.51-1.11)
Quartile 3	269	49	1,999	2.45	0.61 (0.42-0.87)	0.87 (0.58-1.30)	0.80 (0.53-1.21)	1.32 (0.86-2.01)
Quartile 4	269	82	1,638	5.00	1.21 (0.88-1.65)	1.75 (1.22-2.52)	1.50 (1.03-2.18)	1.53 (1.04-2.26)
**Death**								
PH definition 1	938	312	7,754	4.02	2.98 (2.30-3.87)	2.77 (2.12-3.63)	2.00 (1.52-2.64)	1.87 (1.41-2.47)
PH definition 2	733	236	6,082	3.88	2.51 (1.84-3.42)	2.41 (1.76-3.29)	1.85 (1.34-2.55)	1.76 (1.27-2.44)
Continuous per SD increase in rate of TRV	1,076	388	8,718	4.45	1.15 (1.05-1.27)	1.32 (1.22-1.43)	1.19 (1.09-1.31)	1.15 (1.04-1.26)
Quartile 1	269	96	2,153	4.46	Ref	Ref	Ref	Ref
Quartile 2	269	79	2,351	3.36	0.75 (0.55-1.00)	1.13 (0.82-1.56)	1.01 (0.73-1.40)	1.03 (0.74-1.42)
Quartile 3	269	86	2,184	3.94	0.88 (0.66-1.18)	1.56 (1.12-2.16)	1.29 (0.92-1.81)	1.43 (1.02-2.00)
Quartile 4	269	127	2,030	6.25	1.41 (1.08-1.84)	2.47 (1.83-3.34)	1.76 (1.29-2.41)	1.65 (1.20-2.25)
**Composite of Kidney Failure With Replacement Therapy or Death**								
PH definition 1	938	426	6,814	6.25	2.54 (2.00-3.23)	2.34 (1.83-2.98)	1.94 (1.50-2.49)	1.78 (1.37-2.31)
PH definition 2	733	329	5,328	6.18	2.27 (1.72-2.99)	2.16 (1.64-2.87)	1.83 (1.38-2.44)	1.87 (1.39-2.52)
Continuous per SD increase in rate of TRV	1,076	527	7,559	6.97	1.13 (1.03-1.24)	1.43 (1.30-1.57)	1.31 (1.18-1.45)	1.29 (1.17-1.42)
Quartile 1	269	140	1,826	7.67	Ref	Ref	Ref	Ref
Quartile 2	269	116	2,096	5.54	0.73 (0.57-0.93)	1.06 (0.81-1.38)	0.96 (0.74-1.25)	0.95 (0.72-1.24)
Quartile 3	269	114	1,999	5.70	0.75 (0.58-0.96)	1.25 (0.94-1.65)	1.08 (0.81-1.43)	1.42 (1.07-1.90)
Quartile 4	269	157	1,638	9.58	1.24 (0.99-1.56)	2.12 (1.63-2.75)	1.66 (1.26-2.17)	1.68 (1.28-2.20)

*Note:* Model 1 is adjusted for baseline TRV; model 2 is adjusted for the same factors as model 1 and age, sex, diabetes, hypertension, chronic obstructive pulmonary disease, cancer, history of cardiovascular disease, smoking, systolic blood pressure, use of angiotensin-converting enzyme inhibitors/angiotensin receptor blockers, aldosterone antagonists, and diuretics, and ejection fraction. Model 3 is adjusted for the same factors as model 2 and estimated glomerular filtration rate and 24-hour urinary albumin excretion.

Abbreviations: CI, confidence interval; HR, hazard ratio; PH, pulmonary hypertension; py, person-years; SD, standard deviation; TRV, tricuspid regurgitation velocity.

Those with incident PH were also at higher risk of death and a composite of KFRT and death (Table 3), and those with more rapid increases in TRV were at higher risk of KFRT and death (Table 3), including in the complete case analyses (Table S8).

## Discussion

In this study, we demonstrated that the development of PH and higher TRV were associated with an increased risk of KFRT, death, and a composite of the 2 outcomes. Results were consistent when a different definition of PH was used, when the role of left ventricular diastolic dysfunction was examined, when Fine-Gray subdistribution hazard models were used, and when incident PH was examined exclusively. Our results suggest that PH and incident PH are important risk factors for clinically meaningful endpoints.

### PH and Risk of KFRT

Prior studies investigating the relation between PH and kidney outcomes have demonstrated inconsistent results. In a prior study of 2,592 CRIC participants, there was no significant association between PH (defined using an earlier definition of TRV >2.5 m/s or estimated PASP >35 mm Hg) and a composite renal outcome of 50% eGFR decline and KFRT in adjusted models. Conversely, a large Medicare claims-based case-control study demonstrated an increased risk of KFRT for patients with CKD with PH with short durations of follow-up, although this effect was attenuated and no longer significant after 4-5 years of follow-up.^3^ As PH was ascertained in this study based on claims data, it is possible that fewer patients were identified as having PH than in our study, attenuating the effect. Additionally, a large retrospective study of patients with echocardiograms obtained as part of clinical practice demonstrated that higher estimated pulmonary artery pressures were associated with an increased risk of KFRT or 50% decline in eGFR, although these results were potentially biased toward higher pulmonary artery pressures as the echocardiograms were obtained for clinical indications and the study was not a prospective cohort study. Our study adds to the prior CRIC Study by incorporating additional research-grade echocardiograms, having longer follow-up duration (12.1 vs 4.0 years in the prior study) and therefore increasing statistical power by having more outcomes and focusing on KFRT. We also used a more stringent definition of PH of >2.8 m/s, which is consistent with updated guidelines for screening for PH via echocardiogram.^1^^,^^18^

There are multiple reasons why PH may be associated with higher risk of KFRT. First, increasing pressures in the right ventricle may lead to venous congestion, which is now thought to be significant risk for declines in GFR in heart failure.^7^, ^8^, ^9^, ^10^, ^11^ Additionally, PH is a heterogeneous condition, most commonly because of a result of isolated elevations in pulmonary artery pressures (group 1 PH), left ventricular disease (group 2), or lung disease (group 3), and patients with CKD are at risk for each of these conditions. PH is associated with left ventricular failure, which, in its terminal stages, can be associated with declines in blood pressure as well renin-angiotensin-aldosterone system activation, lower renal blood flow, and higher risk of progression of kidney disease. We have attempted to better account for these confounding variables through adjusted analyses, both for left ventricular ejection fraction and diastolic dysfunction, and noted consistent results. We also did not note any overt effect modification and additionally found that our results remain significant without any left ventricular diastolic dysfunction interaction. Additionally, adjusting for chronic obstructive pulmonary disease, one of the few pulmonary comorbid conditions ascertained in CRIC, did not attenuate the relations.

### Development of PH and Risk of Death and Composite Risk of KFRT or Death

Consistent with prior reports, we demonstrated that PH is associated with an increased risk of death.^3^^,^^5^^,^^6^ When the composite of death or KFRT as well as when Fine-Gray subdistribution hazard models were examined, this relation remained consistent. PH may increase CKD patients’ risk of death by increasing the risk of right ventricular failure and sudden cardiac death.^20^ In addition, PH may reflect the severity of multiple comorbid conditions such as acute decompensated heart failure resulting in volume overload, cardiovascular disease, and chronic obstructive pulmonary disease (group 3 PH). We attempted to adjust for these comorbid conditions in our analyses.^21^

In terms of clinical implications, these results call attention to the morbidity and mortality burden associated with PH among patients with CKD. More research is needed into the risk factors for development of PH in CKD so as to engineer treatments that may decrease the risk of PH in this population. For example, one could imagine that closer attention to volume in patients with PH because of left-sided heart disease may disrupt both the renal congestion leading to kidney function declines as well as halt remodeling of pulmonary vasculature that occurs because of ongoing fluid overload.

### Strengths and Limitations

The CRIC cohort is well-suited to analyze this question, given that it is a cohort study (meaning that echocardiograms were not obtained for clinical reasons) with detailed ascertainment of exposures and adjudicated outcomes as well as research-grade echocardiograms performed at prespecified intervals with participants followed for a long duration. The use of time-updated models enabled incorporation of each TRV measurement available.

The main limitation of the study is the use of TRV to define participants as having PH. We have attempted to mitigate this bias by examining TRV as a continuous variable instead, and results were consistent. Ideally, for the diagnosis of PH, participants would undergo a right heart catheterization, the gold-standard test for measurement of pulmonary artery pressures, which also allows for phenotyping patients by whether the PH is because of precapillary, postcapillary, or combined pre- and postcapillary mechanisms. No prospective cohorts of patients with CKD undergoing right heart catheterization exist, however, likely because of the morbidity associated with the procedure. Echocardiography remains the most readily available test for screening for PH, and we note that even after adjusting for diastolic dysfunction and ejection fraction, our results remained consistent. Additionally, as this is an observational analysis, there may be residual confounding from unmeasured variables or imperfect measurement of covariates.

In conclusion, PH and higher TRV were associated with an increased risk of KFRT and death in patients with CKD. Additional research is needed to investigate mechanisms of PH in CKD so as to design therapies and treatments for this common comorbid condition.
