# Supplementary material for: The Association Between Pulmonary Hypertension, End-Stage Kidney Disease, and Death: An Analysis of the Chronic Renal Insufficiency Cohort Study (CRIC)
Source: Kidney Med. 2026 May 12;8(7):101399. doi: 10.1016/j.xkme.2026.101399 (PMC13265864; doi:10.1016/j.xkme.2026.101399)
Supplement: Supplementary File (PDF) — Figure S1-S2; Table S1-S8 [file mmc1.pdf]

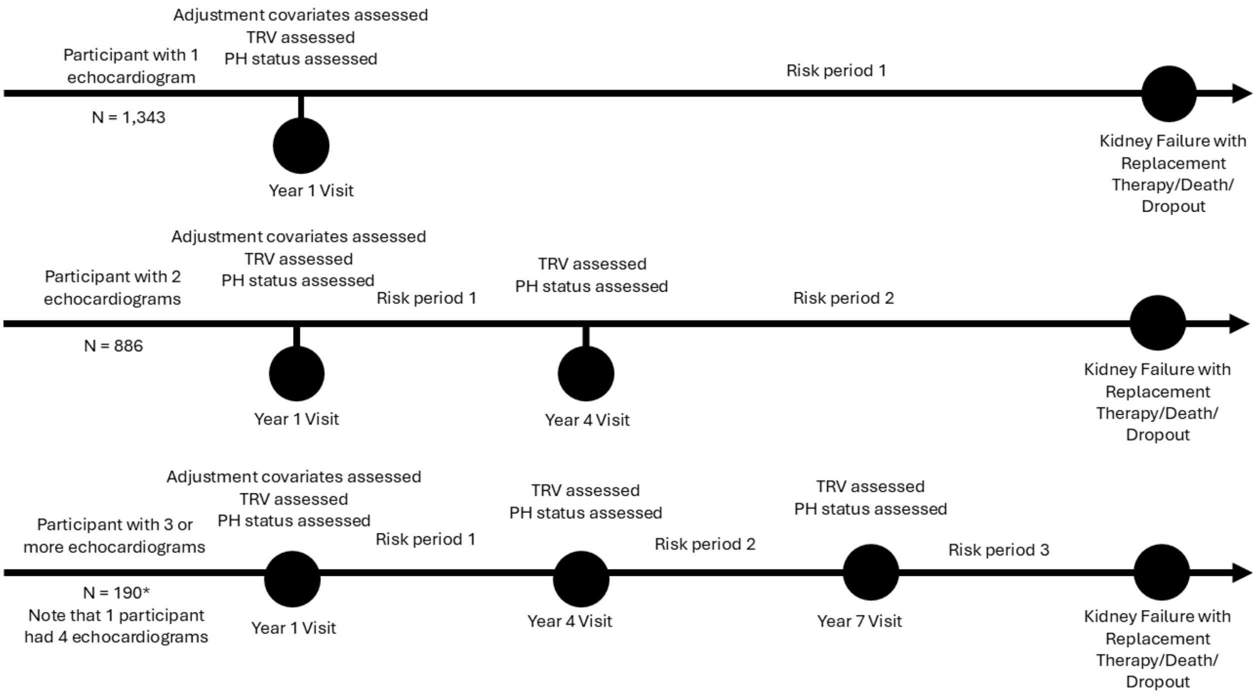

Figure S1. Schematic of Time-Updated Cox Proportional Hazards Model. PH status/TRV was updated at the time of each echocardiogram for an individual participant. Adjustment covariates were ascertained at the time of the first echocardiogram for each participant.

Figure S2: Patient flow diagram for the incident PH analysis

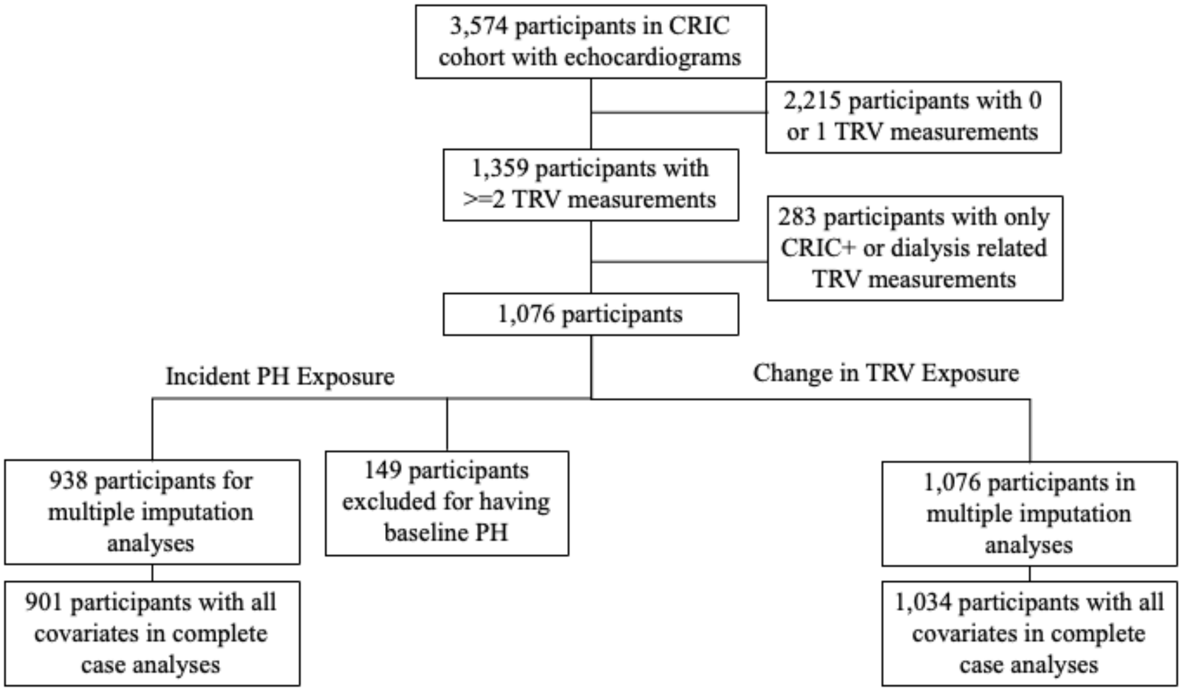

Table S1. Baseline characteristics by number of TRV measurements

Baseline Characteristic	All patients with echocardiograms	Patients with 0 TRV values	Patients with 1 TRV value	Patients with 2 or more TRV values
n	N = 3572	N = 1152	N = 1344	N = 1076
**Demographics**				
Age, years	58.9 (10.9)	56.9 (11.2)	59.2 (11.0)	60.6 (10.0)
Sex, female	1615 (45.2)	482 (41.8)	609 (45.3)	524 (48.7)
Race				
Non-Hispanic white	1507 (42.2)	502 (43.6)	506 (37.6)	499 (46.4)
Non-Hispanic black	1462 (40.9)	473 (41.1)	566 (42.1)	423 (39.3)
Hispanic	464 (13.0)	131 (11.4)	221 (16.4)	112 (10.4)
Other	139 (3.9)	46 (4.0)	51 (3.8)	42 (3.9)
**Vitals**				
Systolic blood pressure, mmHg	127.5 (21.8)	128.0 (22.3)	129.4 (22.5)	124.4 (19.9)
**Comorbidities**				
COPD	173 (4.9)	47 (4.1)	74 (5.5)	52 (4.8)
Diabetes	1791 (50.1)	666 (57.8)	708 (52.7)	417 (38.8)
Hypertension	3170 (88.7)	1025 (89.0)	1221 (90.8)	924 (85.9)
Cardiovascular Disease	1274 (35.7)	419 (36.4)	505 (37.6)	350 (32.5)
Active Smoking	433 (12.1)	151 (13.1)	176 (13.1)	106 (9.9)
Cancer	331 (9.3)	110 (9.5)	127 (9.4)	94 (8.7)
**Laboratory Data**				
eGFR, ml/min/1.73m^2	42.3 (16.2)	41.5 (17.4)	41.0 (16.2)	44.9 (14.7)
Urine albumin excretion, g/24 hours	0.06 [0.01, 0.55]	0.13 [0.01, 0.91]	0.08 [0.01, 0.64]	0.03 [0.01, 0.17]
**Medications**				
ACE inhibitors/ARBs	2451 (68.7)	804 (69.9)	906 (67.5)	741 (68.9)
Aldosterone Antagonist	140 (3.9)	45 (3.9)	55 (4.1)	40 (3.7)
Diuretics	2116 (59.3)	707 (61.5)	826 (61.5)	583 (54.2)
Statins	2115 (59.3)	695 (60.4)	808 (60.2)	612 (56.9)
**Echocardiogram Variables**				
Left ventricular Ejection Fraction (%)	53.9 (8.7)	53.6 (8.3)	53.5 (9.2)	54.8 (8.4)
Baseline TRV m/s	247.9 (35.5)	N/A	249.8 (38.5)	246.2 (32.4)

Data shown as n (%) or mean (SD) or median [25th, 75th percentiles]. Patients who progressed to dialysis prior to the echocardiogram were excluded.

Cardiovascular disease is defined as any of myocardial infarction/revascularization, congestive heart failure, stroke or peripheral vascular disease.

Abbreviations: eGFR = estimated glomerular filtration rate, ACE = angiotensin converting enzyme, ARB = angiotensin receptor blocker, COPD = chronic obstructive pulmonary disease, TRV = tricuspid regurgitation velocity

Table S2. Baseline Characteristics by Quartile of baseline TRV

Quartile of baseline TRV (range, cm/s)	Total	Q1 (160, 220)	Q2 (221, 240)	Q3 (241, 264)	Q4 (265, 411)
n	2419	618	583	610	608
**Demographics**					
Age, years	60.5 (10.6)	58.3 (11.5)	59.7 (10.9)	60.9 (9.9)	63.2 (9.2)
Women	1133 (46.8)	289 (46.8)	257 (44.1)	299 (49.0)	288 (47.4)
Race					
Non-hispanic white	1005 (41.5)	298 (48.2)	256 (43.9)	249 (40.8)	202 (33.2)
Non-hispanic black	989 (40.9)	228 (36.9)	235 (40.3)	244 (40.0)	282 (46.4)
Hispanic	332 (13.7)	70 (11.3)	63 (10.8)	92 (15.1)	107 (17.6)
Other	93 (3.8)	22 (3.6)	29 (5.0)	25 (4.1)	17 (2.8)
**Vitals**					
Systolic blood pressure, mmHg	127.3 (21.7)	122.2 (19.8)	125.8 (19.8)	126.9 (21.3)	134.4 (23.7)
**Comorbidities**					
COPD	140 (5.8)	28 (4.5)	27 (4.6)	33 (5.4)	52 (8.6)
Diabetes	1145 (47.3)	236 (38.2)	255 (43.7)	304 (49.8)	350 (57.6)
Hypertension	2164 (89.5)	517 (83.7)	520 (89.2)	551 (90.3)	576 (94.7)
Cardiovascular disease	881 (36.4)	184 (29.8)	200 (34.3)	210 (34.4)	287 (47.2)
Active Smoking	272 (11.2)	62 (10.0)	71 (12.2)	68 (11.1)	71 (11.7)
Cancer	245 (10.1)	64 (10.4)	64 (11.0)	56 (9.2)	61 (10.0)
**Laboratory Values**					
eGFR, ml/min/1.73m^2	42.0 (16.1)	44.8 (16.4)	43.0 (16.3)	42.1 (16.2)	37.9 (14.7)
Urine albumin excretion, g/24h	0.04 [0.01, 0.39]	0.03 [0.01, 0.29]	0.03 [0.01, 0.33]	0.05 [0.01, 0.41]	0.09 [0.02, 0.67]
**Medications**					
ACE inhibitors/ARBs	1639 (67.8)	401 (64.9)	401 (68.8)	426 (69.8)	411 (67.7)
Aldosterone Antagonist	95 (3.9)	23 (3.7)	22 (3.8)	20 (3.3)	30 (4.9)
Diuretics	1409 (58.3)	297 (48.1)	324 (55.6)	364 (59.7)	424 (69.9)
Statins	1467 (60.7)	339 (54.9)	340 (58.3)	377 (61.8)	411 (67.7)
**Echocardiogram Variables**					
Ejection Fraction, %	53.2 (9.2)	53.8 (7.7)	53.6 (8.5)	53.6 (8.8)	51.9 (11.1)
Baseline TRV cm/s	246.0 (35.1)	208.8 (7.9)	230.3 (5.5)	251.5 (6.7)	293.5 (28.8)

Data shown as n (%) or mean (SD) or median [25th, 75th percentiles]. Cardiovascular disease is defined as any of myocardial infarction/revascularization, congestive heart failure, stroke or peripheral vascular disease
Abbreviations: eGFR = estimated glomerular filtration rate, ACE = angiotensin converting enzyme, ARB = angiotensin receptor blocker, COPD = chronic obstructive pulmonary disease, TRV = tricuspid regurgitation velocity

Table S3. Association of time-updated PH status and TRV with kidney failure with replacement therapy, death, and a composite of kidney failure with replacement therapy or death in the complete case

	N	N Events	Follow-up (years)	Event Rate per 100-py	Unadjusted	Model 1	Model 2
**Kidney Failure with Replacement Therapy**							
**PH**							
PH definition 1	3495	701	19466	3.60	2.23 (1.86, 2.67)	1.79 (1.48, 2.16)	1.62 (1.33, 1.96)
PH definition 2	3060	635	17598	3.61	2.20 (1.84, 2.64)	1.73 (1.43, 2.09)	1.62 (1.34, 1.96)
**TRV**							
Continuous per SD higher TRV	3495	701	19466	3.60	1.35 (1.26, 1.44)	1.24 (1.15, 1.33)	1.19 (1.11, 1.28)
Quartile 1	872	164	5512	2.98	Ref	Ref	Ref
Quartile 2	812	143	4630	3.09	1.04 (0.84, 1.30)	0.98 (0.79, 1.22)	0.97 (0.77, 1.21)
Quartile 3	880	156	5066	3.08	1.00 (0.80, 1.25)	0.89 (0.71, 1.11)	0.94 (0.76, 1.18)
Quartile 4	931	238	4258	5.59	1.91 (1.56, 2.33)	1.53 (1.24, 1.89)	1.41 (1.14, 1.75)
**Death**							
**PH**							
PH definition 1	3495	921	23171	3.97	2.71 (2.34, 3.14)	1.83 (1.57, 2.14)	1.70 (1.46, 1.98)
PH definition 2	3060	799	20397	3.92	2.57 (2.21, 3.00)	1.80 (1.53, 2.11)	1.65 (1.40, 1.93)
**TRV**							
Continuous per SD higher TRV	3495	921	23171	3.97	1.48 (1.40, 1.56)	1.27 (1.19, 1.34)	1.23 (1.16, 1.31)
Quartile 1	872	166	6397	2.59	Ref	Ref	Ref
Quartile 2	812	189	5389	3.51	1.36 (1.11, 1.67)	1.15 (0.94, 1.42)	1.18 (0.96, 1.45)
Quartile 3	880	197	5921	3.33	1.31 (1.07, 1.61)	1.03 (0.84, 1.27)	1.06 (0.86, 1.30)
Quartile 4	931	369.00	5464	6.75	2.80 (2.33, 3.37)	1.78 (1.48, 2.16)	1.71 (1.41, 2.07)
**Composite of Kidney Failure with Replacement Therapy or Death**							
**PH**							
PH definition 1	3495	1268	19466	6.51	2.48 (2.18, 2.83)	1.82 (1.58, 2.09)	1.64 (1.42, 1.88)
PH definition 2	3060	1116	17598	6.34	2.34 (2.05, 2.68)	1.76 (1.53, 2.03)	1.59 (1.38, 1.83)
**TRV**							
Continuous per SD higher TRV	3495	1268	19466	6.51	1.43 (1.36, 1.50)	1.27 (1.20, 1.34)	1.21 (1.15, 1.28)
Quartile 1	872	261	5512	4.74	Ref	Ref	Ref
Quartile 2	812	272	4630	5.87	1.25 (1.06, 1.48)	1.12 (0.95, 1.33)	1.12 (0.94, 1.32)
Quartile 3	880	280	5066	5.53	1.14 (0.97, 1.36)	0.97 (0.82, 1.15)	0.98 (0.83, 1.16)
Quartile 4	931	455	4258	10.69	2.38 (2.04, 2.77)	1.70 (1.45, 1.99)	1.52 (1.30, 1.79)

Model 1 is adjusted for age, sex, diabetes, hypertension, COPD, cancer, history of cardiovascular disease, smoking, systolic blood pressure, ACEi/ARB, aldosterone antagonist, statin and diuretic use and ejection fraction. Model 2 is adjusted for the same factors as Model 1 and eGFR and 24-hour urine albumin excretion. Note that N is representative of the number of person-intervals included in the model and this number is greater than the number of participants in the study as each participant can contribute multiple person-intervals to the model

Abbreviations: HR = hazard ratio, CI = confidence interval, PH = pulmonary hypertension, TRV = tricuspid regurgitation velocity, SD = standard deviation, COPD = chronic obstructive pulmonary disease, ACEi = angiotensin converting enzyme inhibitor, ARB = angiotensin receptor blocker, eGFR = estimated glomerular filtration rate.

Table S4. Association of time-updated PH status and TRV with kidney failure with replacement therapy, death, and a composite of kidney failure with replacement therapy or death with additional adjustments for diastolic dysfunction

	N	N Events	Follow-up (years)	Event Rate per 100-py	Model 2	Model 3	Model 4	P-value
**Kidney Failure with Replacement Therapy**								
PH definition 1	3686	751	20503	3.66	1.60 (1.33, 1.92)	1.60 (1.33, 1.93)	1.73 (1.23, 2.43)	0.23
PH definition 2	3214	680	18439	3.69	1.62 (1.34, 1.95)	1.64 (1.36, 1.98)	1.73 (1.24, 2.43)	0.43
Continuous per SD of TRV	3686	751	20503	3.66	1.20 (1.12, 1.28)	1.20 (1.12, 1.28)	1.23 (1.10, 1.38)	0.12
Quartile 1	919	175	5803	3.02	Ref	Ref	Ref	0.32
Quartile 2	848	151	4869	3.10	0.95 (0.76, 1.19)	0.95 (0.76, 1.19)	1.02 (0.68, 1.54)	
Quartile 3	933	166	5338	3.11	0.97 (0.78, 1.21)	0.97 (0.78, 1.20)	1.14 (0.75, 1.73)	
Quartile 4	986	259	4493	5.77	1.43 (1.16, 1.75)	1.43 (1.16, 1.75)	1.57 (1.09, 2.25)	
**Death**								
PH definition 1	3686	978	24486	3.99	1.68 (1.45, 1.95)	1.70 (1.46, 1.98)	1.51 (1.12, 2.05)	0.81
PH definition 2	3214	844	22110	3.82	1.63 (1.39, 1.91)	1.66 (1.42, 1.94)	1.33 (0.98, 1.82)	0.32
Continuous per SD of TRV	3686	978	24486	3.99	1.23 (1.16, 1.30)	1.23 (1.17, 1.31)	1.21 (1.08, 1.35)	0.85
Quartile 1	919	175	6759	2.59	Ref	Ref	Ref	0.07
Quartile 2	848	198	5681	3.49	1.19 (0.97, 1.45)	1.19 (0.97, 1.46)	1.27 (0.81, 1.98)	
Quartile 3	933	210	6243	3.36	1.04 (0.85, 1.27)	1.03 (0.84, 1.27)	1.64 (1.08, 2.51)	
Quartile 4	986	395	5803	6.81	1.64 (1.36, 1.97)	1.65 (1.38, 1.99)	1.76 (1.20, 2.58)	
**Composite of Kidney Failure with Replacement Therapy or Death**								
PH definition 1	3686	1352	20503	6.59	1.60 (1.40, 1.84)	1.61 (1.41, 1.84)	1.64 (1.28, 2.09)	0.33
PH definition 2	3214	1186	18439	6.43	1.57 (1.37, 1.81)	1.59 (1.38, 1.83)	1.57 (1.22, 2.02)	0.42
Continuous per SD of TRV	3686	1352	20503	6.59	1.21 (1.15, 1.27)	1.21 (1.15, 1.27)	1.21 (1.11, 1.32)	0.34
Quartile 1	919	278	5803	4.79	Ref	Ref	Ref	0.59
Quartile 2	848	286	4869	5.87	1.10 (0.93, 1.30)	1.10 (0.93, 1.30)	1.03 (0.74, 1.42)	
Quartile 3	933	298	5338	5.58	0.99 (0.84, 1.17)	0.99 (0.84, 1.17)	1.17 (0.85, 1.61)	
Quartile 4	986	490	4493	10.91	1.49 (1.28, 1.74)	1.50 (1.28, 1.75)	1.48 (1.12, 1.97)	

Model 2 is adjusted age, sex, diabetes, hypertension, COPD, cancer, history of cardiovascular disease, smoking, systolic blood pressure, ACEi/ARB, aldosterone antagonist, statin, and diuretic use, ejection fraction, eGFR and 24-hour urine albumin excretion. Model 3 is adjusted for the same factors as model 2 with the addition of diastolic dysfunction. Model 4 is adjusted for the same factors as model 3 with an interaction term between diastolic dysfunction and the exposure of interest. P-values represent the p-value for the interaction term of Model 4. Note that N is representative of the number of person-intervals included in the model and this number is greater than the number of participants in the study as each participant can contribute multiple person-intervals to the model

Abbreviations: HR = hazard ratio, CI = confidence interval, PH = pulmonary hypertension, TRV = tricuspid regurgitation velocity, SD = standard deviation, COPD = chronic obstructive pulmonary disease, ACEi = angiotensin converting enzyme inhibitor, ARB = angiotensin receptor blocker, eGFR = estimated glomerular filtration rate.

Table S5. Fine-Gray models for the association of time-updated PH status and TRV with kidney failure with replacement therapy accounting for the competing risk of death

	N	N KFRT Events	Follow-up (years)	Event Rate per 100-py	Unadjusted	Model 1	Model 2
**PH**							
PH definition 1	3495	701	19466	3.60	1.78 (1.50, 2.11)	1.57 (1.31, 1.87)	1.38 (1.16, 1.63)
PH definition 2	3060	635	17598	3.61	1.80 (1.52, 2.13)	1.54 (1.28, 1.85)	1.37 (1.16, 1.63)
**TRV**							
Continuous per SD higher TRV	3495	701	19466	3.60	1.23 (1.16, 1.31)	1.17 (1.10, 1.26)	1.08 (1.02, 1.15)
Quartile 1	872	164	5512	2.98	Ref	Ref	Ref
Quartile 2	812	143	4630	3.09	0.97 (0.78, 1.20)	0.95 (0.77, 1.18)	0.93 (0.76, 1.14)
Quartile 3	880	156	5066	3.08	0.99 (0.80, 1.22)	0.95 (0.77, 1.18)	0.94 (0.77, 1.16)
Quartile 4	931	238	4258	5.59	1.58 (1.31, 1.90)	1.39 (1.14, 1.70)	1.25 (1.03, 1.51)

Model 1 is adjusted for age, sex, diabetes, hypertension, COPD, cancer, history of cardiovascular disease, smoking, systolic blood pressure, ACEi/ARB, aldosterone antagonist, statin and diuretic use and ejection fraction. Model 2 is adjusted for the same factors as Model 1 and eGFR and 24-hour urine albumin excretion. Note that N is representative of the number of person-intervals included in the model and this number is greater than the number of participants in the study as each participant can contribute multiple person-intervals to the model.

Abbreviations: HR = hazard ratio, CI = confidence interval, PH = pulmonary hypertension, TRV = tricuspid regurgitation velocity, SD = standard deviation, COPD = chronic obstructive pulmonary disease, ACEi = angiotensin converting enzyme inhibitor, ARB = angiotensin receptor blocker, eGFR = estimated glomerular filtration rate.

Table S6. Participant characteristics by PH category in the subset of patients with 2 echocardiograms in patients with incident PH or who never develop PH via definition 1.

Patient Characteristic	Total	Incident PH	Never PH	P-value
n	938	119	819	
**Demographics**				
Age, years	64.2 (10.1)	68.2 (8.1)	63.7 (10.3)	<0.001
Sex, female	457 (48.7)	56 (47.1)	401 (49.0)	0.77
Race				0.03
Non-Hispanic white	449 (47.9)	42 (35.3)	407 (49.7)	
Non-Hispanic black	360 (38.4)	58 (48.7)	302 (36.9)	
Hispanic	93 (9.9)	14 (11.8)	79 (9.6)	
Other	36 (3.8)	5 (4.2)	31 (3.8)	
**Vitals**				
Systolic blood pressure, mmHg	126.5 (20.5)	135.2 (26.2)	125.3 (19.2)	<0.001
**Comorbidities**				
Chronic Obstructive Pulmonary Disease	76 (8.1)	14 (11.8)	62 (7.6)	0.17
Diabetes	397 (42.3)	66 (55.5)	331 (40.4)	0.003
Hypertension	857 (91.4)	116 (97.5)	741 (90.5)	0.02
Cardiovascular disease	359 (38.3)	67 (56.3)	292 (35.7)	<0.001
Active Smoking	79 (8.4)	9 (7.6)	70 (8.5)	0.85
Cancer	154 (16.4)	22 (18.5)	132 (16.1)	0.60
**Laboratory Values**				
eGFR, ml/min/1.73m^2	43.7 (18.4)	36.4 (16.7)	44.7 (18.4)	<0.001
Urine albumin excretion, g/24h	0.03 [0.01, 0.14]	0.04 [0.01, 0.16]	0.02 [0.01, 0.14]	0.15
**Medications**				
ACE inhibitors/ARBs	637 (67.9)	81 (68.1)	556 (67.9)	1.00
Aldosterone Antagonist	41 (4.4)	8 (6.7)	33 (4.0)	0.27
Diuretics	471 (50.2)	81 (68.1)	390 (47.6)	<0.001
Statins	575 (61.3)	84 (70.6)	491 (60.0)	0.03
**Echocardiogram Variables**				
Ejection Fraction (%)	50.3 (8.8)	47.6 (11.0)	50.7 (8.4)	<0.001
Baseline TRV cm/s	236.9 (22.5)	247.9 (21.0)	235.3 (22.2)	<0.001

Data shown as n (%) or mean (SD) or median [25th, 75th percentiles]. All characteristics are derived at or before the time of the second echocardiogram with the exception of baseline TRV. Cardiovascular disease is defined as any of myocardial infarction/revascularization, congestive heart failure, stroke or peripheral vascular disease. Abbreviations: eGFR = estimated glomerular filtration rate, ACE = angiotensin converting enzyme, ARB = angiotensin receptor blocker, COPD = chronic obstructive pulmonary disease, TRV = tricuspid regurgitation velocity

Table S7. Participant characteristics by Quartile of Rate of TRV Change

Quartile of Rate of TRV Change (range, cm/s/year)	Total	Q1 (-49.66, -5.58)	Q2 (-5.57, 1.03)	Q3 (1.04, 7.53)	Q4 (7.54, 94.97)
n	1076	269	269	269	269
**Demographics**					
Age, years	64.5 (10.0)	64.0 (10.3)	64.2 (10.4)	64.0 (10.0)	65.6 (9.4)
Sex, female	524 (48.7)	115 (42.8)	131 (48.7)	146 (54.3)	132 (49.1)
Race					
Non-Hispanic white	499 (46.4)	132 (49.1)	134 (49.8)	121 (45.0)	112 (41.6)
Non-Hispanic black	423 (39.3)	98 (36.4)	91 (33.8)	113 (42.0)	121 (45.0)
Hispanic	112 (10.4)	28 (10.4)	33 (12.3)	24 (8.9)	27 (10.0)
Other	42 (3.9)	11 (4.1)	11 (4.1)	11 (4.1)	9 (3.3)
**Vitals**					
SBP, mmHg	127.3 (21.0)	126.6 (19.2)	126.8 (20.7)	123.9 (19.4)	132.1 (23.5)
**Comorbidities**					
COPD	100 (9.3)	36 (13.4)	17 (6.3)	25 (9.3)	22 (8.2)
Diabetes	477 (44.3)	129 (48.0)	111 (41.3)	106 (39.4)	131 (48.7)
Hypertension	992 (92.2)	247 (91.8)	245 (91.1)	245 (91.1)	255 (94.8)
Cardiovascular disease	424 (39.4)	103 (38.3)	94 (34.9)	103 (38.3)	124 (46.1)
Active Smoking	89 (8.3)	23 (8.6)	20 (7.4)	20 (7.4)	26 (9.7)
Cancer	176 (16.4)	38 (14.1)	44 (16.4)	43 (16.0)	51 (19.0)
**Laboratory Values**					
eGFR, ml/min/1.73m^2	42.6 (18.1)	41.9 (17.7)	44.2 (18.4)	45.7 (17.6)	38.9 (18.1)
Urine albumin excretion, g/24h	0.03 [0.01, 0.17]	0.05 [0.01, 0.43]	0.02 [0.01, 0.13]	0.02 [0.01, 0.09]	0.03 [0.01, 0.21]
**Medications**					
ACE inhibitors/ARBs	732 (68.0)	184 (68.4)	182 (67.7)	186 (69.1)	180 (66.9)
Aldosterone Antagonist	47 (4.4)	12 (4.5)	8 (3.0)	17 (6.3)	10 (3.7)
Diuretics	569 (52.9)	152 (56.5)	132 (49.1)	131 (48.7)	154 (57.2)
Statins	663 (61.6)	173 (64.3)	168 (62.5)	156 (58.0)	166 (61.7)
**Echocardiogram Variables**					
Ejection Fraction (%)	50.2 (9.0)	50.7 (8.6)	50.8 (8.4)	49.6 (8.4)	49.4 (10.5)
Baseline TRV cm/s	245.7 (32.3)	272.4 (32.7)	243.1 (27.0)	233.8 (23.8)	233.5 (28.5)

Rate of TRV change calculated as (first TRV – last TRV) / years between echocardiograms for a given patient. All characteristics are derived at or before the time of the second echocardiogram with the exception of baseline TRV. Data shown as n (%) or mean (SD) or median [25th, 75th percentiles]. Cardiovascular disease is defined as any of myocardial infarction/revascularization, congestive heart failure, stroke or peripheral vascular disease. Abbreviations: eGFR = estimated glomerular filtration rate, ACE = angiotensin converting enzyme, ARB = angiotensin receptor blocker, COPD = chronic obstructive pulmonary disease, TRV = tricuspid regurgitation velocity

Table S8. Association of incident pulmonary hypertension/rate of TRV change with kidney failure with replacement therapy, death and a composite of kidney failure with replacement therapy or death using the complete case

**Exposure**	**N**	**N Events**	**Follow-up (years)**	**Event Rate per 100-py**	**Unadjusted HR (95%CI)**	**Model 1 HR (95%CI)**	**Model 2 HR (95%CI)**	**Model 3 HR (95%CI)**
**Kidney Failure with Replacement Therapy**								
PH definition 1	901	203	6516	3.12	2.18 (1.53, 3.11)	2.09 (1.45, 2.99)	2.03 (1.39, 2.96)	1.81 (1.21, 2.70)
PH definition 2	705	170	5097	3.33	2.23 (1.53, 3.24)	2.15 (1.47, 3.15)	2.04 (1.38, 3.03)	1.98 (1.30, 3.04)
Continuous per SD increase in TRV	1034	249	7247	3.44	1.14 (0.99, 1.30)	1.37 (1.19, 1.57)	1.33 (1.14, 1.54)	1.36 (1.19, 1.56)
Quartile 1	258	74	1740	4.25	Ref	Ref	Ref	Ref
Quartile 2	260	50	2021	2.47	0.60 (0.42, 0.85)	0.75 (0.51, 1.11)	0.69 (0.47, 1.02)	0.76 (0.51, 1.13)
Quartile 3	258	48	1906	2.52	0.61 (0.42, 0.87)	0.83 (0.55, 1.24)	0.76 (0.50, 1.15)	1.32 (0.86, 2.01)
Quartile 4	258	77	1580	4.87	1.14 (0.83, 1.57)	1.60 (1.11, 2.32)	1.38 (0.94, 2.04)	1.56 (1.04, 2.34)
**Death**								
PH definition 1	901	304	7423	4.10	2.84 (2.18, 3.70)	2.66 (2.03, 3.49)	1.94 (1.47, 2.56)	1.82 (1.37, 2.41)
PH definition 2	705	231	5821	3.97	2.41 (1.76, 3.29)	2.32 (1.69, 3.17)	1.82 (1.32, 2.51)	1.74 (1.26, 2.40)
Continuous per SD increase in TRV	1034	377	8355	4.51	1.14 (1.04, 1.26)	1.32 (1.21, 1.43)	1.20 (1.09, 1.32)	1.16 (1.06, 1.27)
Quartile 1	258	93	2058	4.52	Ref	Ref	Ref	Ref
Quartile 2	260	79	2264	3.49	0.76 (0.57, 1.03)	1.18 (0.85, 1.62)	1.04 (0.75, 1.45)	1.08 (0.78, 1.50)
Quartile 3	258	83	2088	3.97	0.88 (0.65, 1.18)	1.57 (1.12, 2.19)	1.31 (0.93, 1.85)	1.47 (1.04, 2.07)
Quartile 4	258	122	1944	6.27	1.39 (1.06, 1.82)	2.53 (1.86, 3.45)	1.87 (1.35, 2.59)	1.81 (1.31, 2.50)
**Composite of Kidney Failure with Replacement Therapy or Death**								
PH definition 1	901	412	6516	6.32	2.47 (1.94, 3.15)	2.29 (1.78, 2.93)	1.90 (1.47, 2.46)	1.83 (1.41, 2.38)
PH definition 2	705	319	5097	6.26	2.20 (1.67, 2.92)	2.11 (1.59, 2.80)	1.81 (1.36, 2.41)	1.90 (1.41, 2.54)
Continuous per SD increase in TRV	1034	508	7247	7.01	1.12 (1.02, 1.24)	1.42 (1.29, 1.57)	1.31 (1.18, 1.46)	1.32 (1.20, 1.46)
Quartile 1	258	136	1740	7.82	Ref	Ref	Ref	Ref
Quartile 2	260	113	2021	5.59	0.72 (0.56, 0.92)	1.03 (0.79, 1.35)	0.93 (0.71, 1.22)	0.97 (0.73, 1.27)
Quartile 3	258	110	1906	5.77	0.74 (0.58, 0.95)	1.21 (0.91, 1.60)	1.04 (0.78, 1.39)	1.42 (1.06, 1.90)
Quartile 4	258	149	1580	9.43	1.20 (0.95, 1.52)	2.01 (1.54, 2.63)	1.60 (1.21, 2.12)	1.75 (1.33, 2.32)

Model 1 is adjusted for baseline TRV, Model 2 is adjusted for the same factors as model 1 and age, sex, diabetes, hypertension, COPD, cancer, history of cardiovascular disease, smoking, systolic blood pressure, ACEi/ARB, aldosterone antagonist and diuretic use and ejection fraction. Model 3 is adjusted for the same factors as model 2 and eGFR and 24-hour urine albumin excretion.

Abbreviations: HR = hazard ratio, CI = confidence interval, PH = pulmonary hypertension, TRV = tricuspid regurgitation velocity, SD = standard deviation, COPD = chronic obstructive pulmonary disease, ACEi = angiotensin converting enzyme inhibitor, ARB = angiotensin receptor blocker, eGFR = estimated glomerular filtration rate.
